# Topical cream with essential oils, zinc and salicylic acid reduces pruritus and skin lesion scores in pruritic dogs

**DOI:** 10.1111/avj.70048

**Published:** 2025-12-08

**Authors:** D Prescott, A Stewart, A Schoep, A Herndon

**Affiliations:** ^1^ School of Veterinary Science, University of Queensland Gatton Queensland 4343 Australia

**Keywords:** atopy, dermatitis, therapeutic

## Abstract

**Objectives:**

To assess the efficacy of an essential oil, salicylic acid and zinc‐based cream in the relief of pruritus not secondary to infectious pyoderma or ectoparasites and associated dermatological lesions.

**Methods:**

Forty‐one client‐owned, otherwise healthy, dogs with chronic, noninfectious pruritus were enrolled in a double‐blinded, placebo‐controlled, randomised clinical trial. Dogs were assigned to receive topical treatment using either the study or placebo cream daily for 14 days. Owners recorded pruritus scores using a visual scale (Pruritus Analog Visual Scale [PVAS]) in a daily diary. Severity of skin lesions was quantified before and after the trial using the Canine Atopic Dermatitis Extent and Severity Index – 4th Generation (CADESI‐4) rubric. Baseline and post‐trial blood counts and serum biochemistries were used to assess health and screen for any evidence of toxicity secondary to cream application.

**Results:**

Fourteen‐day course of treatment with the study cream was associated with 1.75x greater reduction in pruritus score compared to placebo. The reduction in pruritus was greater in the treatment versus placebo groups starting at day 9 of treatment and continued through day 14. Visible skin lesions improved with treatment but did not improve with placebo. Quality of life scores improved in both groups, but improvement was greater in the treatment group.

**Clinical Significance:**

The topical cream used in this study was a safe and effectives complementary treatment for the relief of pruritus and dermatological lesions in dogs.

**Abbreviations**
CADESI‐4: Canine Atopic Dermatitis Extent and Severity Index – 4th Generation
PVAS: Pruritus Analog Visual Scale

Dermatologic conditions including pruritus and inflammatory skin lesions are common presenting complaints at veterinary clinics globally. Pruritus or dermatological complaints are reported to account for between 2.1% and 7.01% of dogs presenting to small animal practice.^1^, ^2^, ^3^ Non‐infectious causes of dermatitis and pruritus such as atopic dermatitis are common, with an estimated prevalence of 10%–15% of the pet dog population.^4^ Atopic dermatitis is a chronic condition often requiring life‐long management of clinical signs.

Current treatment protocols commonly employ antipruritic drugs such as glucocorticoids, antihistamines, immunoregulators or IL‐31 pathway inhibition.^5^, ^6^ Each therapeutic option has a unique risk and benefit profile for each patient. Some dogs are easy to administer oral medications, whereas others are challenging for owners to ensure proper swallowing. Topical treatments may be practical for some dogs, but adequate coverage may be difficult in dogs with large skin areas requiring treatment. In order to maximise benefits and limit risks, it is often necessary to combine therapies.^7^

Antihistamines are inconsistently effective in controlling pruritus in dogs but are occasionally useful in the acute flare‐up period.^8^ Oral ciclosporine has been shown to reduce pruritus in a high proportion of atopic dogs in one study, but was associated with a relatively slow onset of action.^9^ IL‐31 pathway inhibitors oclacitinib and lokivetmab have been demonstrated to have similar or greater antipruritic efficacy to ciclosporin with few adverse events.^9^, ^10^ Glucocorticoids are usually effective for acute flare‐ups and may be effective for longer‐term use if the dose is reduced to an effective anti‐itching dose without adverse side effects.^8^ Adverse side effects are common with chronic glucocorticoid use and were noted in 90% of dogs treated orally with anti‐inflammatory doses of prednisolone after only 14 days of treatment.^11^ Topical application of hydrocortisone aceponate as a spray has been shown to improve skin lesions and pruritus in the acute (2 week) phase and to have similar efficacy and tolerance as compared to oral ciclosporin when used over 12 weeks.^12^, ^13^

There is increasing global interest in ‘natural‐based’ products in a veterinary care setting, with a corresponding trend in research of nontraditional therapies.^14^ Although multifactorial, the reasons underpinning such a movement are clients' desire for efficacious, economical, alternative solutions, even when other registered veterinary pharmaceuticals are available.^15^ Over 90 essential oils have been identified as having a role in treating dermatological conditions,^16^ and several medicinal plants have been suggested as promising therapeutic tools specifically for use in canine dermatology.^17^

The objective of this clinical trial was to assess the efficacy of an essential oil, salicylic acid and zinc‐based topical cream on the reduction of noninfectious pruritus and skin lesions in client‐owned dogs. We hypothesised that the application of the cream would result in a significant reduction in owner‐scored pruritus, as well as veterinarian‐scored skin lesions after two weeks of daily treatment.

## Materials and methods

### Study design and inclusion criteria

A double‐blind, placebo‐controlled and randomised study design was used to test the treatment hypothesis. The project was approved by the University of Queensland Office of Research Integrity animal (2022/AE000800) and human (2023/HE000037) ethics.

Local clients with client‐owned dogs over 12 months old were recruited through social media and local ads. Dogs must be nonpregnant, lactating or intended for breeding and able to observe the washout period for other treatments to participate. Washout periods are defined as one week since baths with chlorhexidine shampoo or treatment with antihistamines, non‐steroidal anti‐inflammatory drugs, antibiotics, short‐acting corticosteroids (hydrocortisone, prednisolone) or oclacitinib. Ciclosporin, lokivetmab and long‐acting corticosteroids require a 4‐week washout period. A definitive diagnosis of atopic dermatitis was not required for enrolment.

Dogs enrolling in the study were required to have received afoxolaner (Nexgard™, Boehringer Ingelheim Animal Health, Duluth, GA), fluralaner (Bravecto™, Merck & Co, Inc, Rahway, NJ, USA) or sarolaner (Simparica Trio™, Zoetis Services LLC, Parsippany, NJ, USA) within the four weeks before their first appointment. If ectoparasite prophylaxis was not administered within that period, owners were provided with a NexGard chew and instructed to administer the treatment at least seven days before their first appointment. Dogs were excluded from the study if pruritus improved after treatment with an ectoparasiticide.

Additional exclusion criteria included: being clinically unwell, haematological or serum biochemical evidence of systemic disease, evidence of an infectious pyoderma or being unable to be safely restrained for blood collection without sedation.

Two groups (treatment or placebo) were randomly allocated *pre ho*c using the randomisation function in Excel® (Microsoft Office 360, 2023) by a single member of the research team not involved in patient recruitment, examination or data collection. Dogs were assigned to preallocated groups based on the order the dogs enrolled in the study during the first visit. Owners and investigators remained blinded to treatment groups until all dogs in each group completed the 2‐week protocol and all data were analysed.

### Cream ingredients

The treatment and placebo creams were provided by the manufacturer and prepared within one month of initiating the trial. They were delivered in identical, opaque tubs differentiated by only the letters ‘A’ or ‘B’. Ingredients used for the treatment and placebo creams are listed in Table 1. Zinc oxide and salicylic acid are considered the principal active ingredients in the treatment cream. Ingredients in the placebo base were not identical to those of the treatment cream, but were constructed to mimic the texture and consistency of the active product. Fragrance was added to the placebo to provide a scent to the cream and limit the chance of owners becoming unblinded from the absence of a scent.

**Table 1:** Ingredient lists for the treatment and placebo‐controlled creams

Cream	Ingredient	Minimum	Content
Treatment	Mineral oil		
	Paraffin wax		
	Vegetable oils		Wheatgerm, sweet almond, jojoba seed, avocado, apricot kernel
	Zinc oxide	2.5%	
	Salicylic acid	1.8%	
	Herbal complex	<3.0%	Proprietary blend of 13 essential oils
	Calendula extract	<1.0%	
	Bergamot oil	<1.0%	
	No preservative		
Placebo	Water	<77%	
	Rice bran oil	8.0%	
	Emulsifying wax	7.0%	
	Glycerine	3.0%	
	Stearic acid	2.0%	
	Phenonip preservative	<1%	
	Xanthan gum	0.5%	
	Fragrance	0.5%	
	Disodium EDTA	0.5%	
	Vitamin E	0.3%	

EDTA, ethylenediaminetetraacetic acid.

### Study procedure

Dogs and their owners attended two appointments 14 days apart. At the initial appointment, a complete history was taken, based on the Canine Atopic Dermatitis Research and Diagnosis Questionnaire (cAD‐RQ).^18^ This was supplemented with questions designed to quantify the number of atopic criteria exhibited by the dog.^19^ At both appointments (Days 0 and 14), owners completed a quality of life (QoL) questionnaire.^20^, ^21^ Investigators performed a basic physical examination, collected blood for complete blood count and serum biochemistry and scored skin lesions using the Canine Atopic Dermatitis Extent and Severity Index – 4th Generation (CADESI‐4) tool.^22^

Owners were instructed to apply the cream once per day on areas of affected or itchy skin, as identified for each individual dog during the initial consultation (e.g. axilla, feet, inguinal regions). Owners were instructed to continue applying the cream to the same areas of the body daily for the entire 2‐week trial period, irrespective of whether they considered the lesion or pruritus to have resolved in that area. Owners were provided with a paper‐based diary to record Pruritus Analog Visual Scale (PVAS) scores daily and note any unusual or adverse events.

### Outcomes

PVAS scores were recorded between a range of 0 (not itchy) and 10 (maximally itchy).^23^, ^24^ Successful treatment was defined as a reduction of ≥2 points on the scale, which would correlate to the reduction of severity by one or more categories (normal or very mild/mild/moderate/severe/very severe).^24^, ^25^ Complete remission was defined by a final PVAS of ≤1.9.^24^

The same investigator used the CADESI‐4 tool to quantify skin lesions at both appointments (Day 1 and 14). The CADESI‐4 scoring tool was developed specifically for dogs with atopic dermatitis but has been used to quantify skin lesion severity in similar studies in which a definitive diagnosis of atopic dermatitis was part of the inclusion criteria.^26^, ^27^

The CADESI‐4 scale allocates scores for erythema, lichenification and excoriations and/or alopecia to 20 different body areas which are then totalled. Totalled scores range from 0 (no lesions) to 180 (maximum lesion score). Scores were further categorised into grades: normal (<10), mild (10–34), moderate (35–59) or severe (>60).^22^

Owners completed the QoL questionnaire at each visit (Day 1 and 14), considering their experiences over the previous seven‐day period. The QoL questionnaire is validated specifically for dogs with skin disease and measures 15 questions on a scale of 0 (least negative effect) to 3 (most negative effect). The first question gives an overall measure of perceived severity, whereas the remaining questions are evenly divided into categories affecting the quality of life for the dog (dog‐QoL) and the owner(s) (owner‐QoL). This results in an overall score between 0 (best QoL) and 42 (worst QoL) for the last 14 questions combined.^20^, ^21^

At day 7 and 14, owners were asked to report on the ease of use of the creams and the dog's behavioural response to treatment: ‘Over the last week, how easy would you say it has been to use this product on your dog?’ (Very easy/easy/fair/hard/very hard); and ‘Over the last week, how well did your dog tolerate the treatment?’ (Enjoyed it/Not bothered/Tolerated/Disliked/Avoided).

Owners were asked to report any adverse events in the diary (vomiting, diarrhoea, self‐trauma)and discontinue application if concerns arose. Complete blood counts and serum biochemistry were collected at Day 1 and Day 14 to assess for any changes over the 14‐day trial period.

### Statistical analysis

Dogs which did not satisfactorily complete the 2‐week trial period, including attending both veterinary visits, were excluded from the subsequent data analysis. Data analysis was performed using commercial software (R, version 4.31, Vienna, Austria; RStudio, version 2023.09, Boston, MA; and the Tidyverse package, version 2.0.0). Groups were compared using Pearson's Chi‐squared tests for categorical variables or two‐sample t‐tests (Student's t‐test or Welch's t‐test) for continuous variables. Within‐group comparisons across timepoints were calculated using paired t‐tests and a corrected linear mixed effect model was used to evaluate changes over time. Significance was set at P < 0.05. Categorical data were presented as integers; continuous variables were presented as mean ± standard deviation.

## Results

### Participants

Forty‐six dogs were enrolled in the study. Two dogs were lost to follow‐up, two dogs were removed after the owner switched creams between dogs, and one owner withdrew due to a dislike of the fragrance of the cream. Forty‐one dogs were included in the data analysis. Eighteen dogs were allocated to the treatment group, and 23 dogs to the placebo group (Table 2). A wide range of breeds was evenly represented; however since no combination of breed and treatment group occurred more than twice, statistical analysis was not performed (Table 3).

**Table 2:** Signalment and scoring parameters for 41 dogs at the start of the trial

Parameter	Treatment (n = 18)	Placebo (n = 23)	P value
Age (years)	5.64 ± 3.37	4.93 ± 3.18	0.5
Sex			0.17
Female	11	8	
Male	7	15	
Desexed			1.00
Yes	16	20	
No	2	3	
Weight (kg)	21.5 ± 13.50	16.0 ± 9.50	0.2
Coat length			0.9
Short	12	14	
Medium	4	8	
Long	2	1	

Continuous variables are displayed as mean ± standard deviation. Categorical variables are displayed as the number of dogs (n).

**Table 3:** Breeds for n = 41 dogs completing the study, by treatment group

Breed	Treatment (n)	Placebo (n)	Total (n)
Crossbreed <10 kg	2	3	5
Crossbreed 10–20 kg	0	3	3
Crossbreed 20–30 kg	2	2	4
American Staffordshire terrier	1	0	1
Australian cattle dog	0	1	1
British Bulldog	0	1	1
Bull Arab	1	0	1
Bull Mastiff	1	0	1
Cavoodle	0	1	1
Dachshund	0	2	2
English Staffordshire terrier	0	2	2
French bulldog	2	0	2
Greyhound	1	0	1
German Shepherd	0	1	1
Great Dane	1	0	1
Husky	1	0	1
Koolie	0	1	1
Labradoodle	0	1	1
Labrador	2	0	2
Maltese	1	1	2
Miniature fox terrier	1	0	1
Pug	1	2	3
Staffordshire terrier	2	0	2
West Highland white terrier	0	1	1

All dogs described as crossbreeds have been simplified into weight brackets.

### Underlying diagnoses

The questionnaire data from the first appointment lacked historical information for some dogs, preventing accurate diagnosis. Although some dogs met the criteria for atopic dermatitis outlined by Favrot et al, insufficient historical data hindered the answer to questions in others. For instance, owners could not clearly articulate the onset of pruritus, lesion distribution or consistency of ectoparasite treatment. Moreover, most dogs had not completed an appropriate elimination diet trial to rule out food allergic dermatitis. No dogs had any evidence of deep pyoderma (ulcerations, crusting lesions, sanguinous or purulent discharge). Skin impression cytology was performed on two dogs with moderate skin changes, and in both cases, only occasional cocci organisms and no neutrophilic population of cells were noted.

### Pruritus scoring

**Figure 1:**
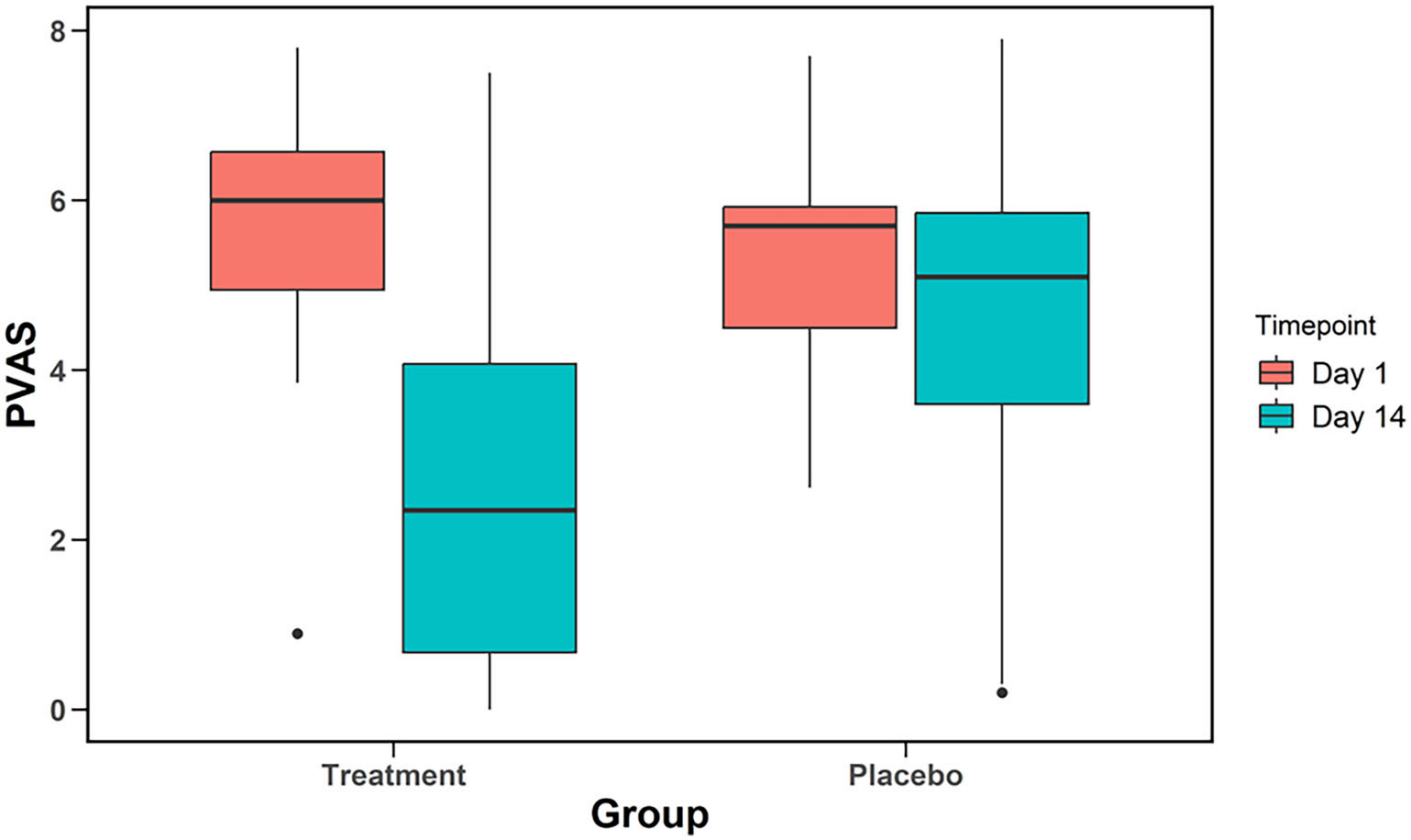
Comparison of pretrial (Day 1) and post‐trial (Day 14) pruritus scores (Pruritus Analog Visual Scale [PVAS]) in the treatment and placebo groups. When comparing Day 14 to Day 1, the treatment group showed a significant decrease (P < 0.001). The placebo group also declined (P = 0.002), but to a much lesser extent.

At Day 1, no difference was detected in PVAS scores between the groups (P = 0.67). The mean PVAS of the treatment group was 5.66 ± 1.70, and the mean PVAS of the placebo group was 5.44 ± 1.55. At the conclusion of the trial (Day 14), the PVAS of both the treatment and placebo groups had significantly decreased (Table 4). Owners of dogs in the treatment group reported a mean reduction of −2.88 whereas the placebo group only decreased −1.13 (P < 0.001 and P = 0.008 respectively). At Day 14, the mean PVAS of the treatment group was 2.78 ± 2.22 and this was significantly lower than that of the placebo group which had a mean of 4.31 ± 2.14 (P = 0.03). When comparing the mean difference between groups from Day 1 to Day 14, there was a 1.75x greater reduction in PVAS in the treatment group compared to the placebo group (P = 0.007, Figure 1).

**Table 4:** Mean Pruritus Analog Visual Scale (PVAS) scores from Day 1 and Day 14 in treatment and control dogs

	Time point	Mean ± SD	P value
Treatment	Day 1	5.66 ± 1.70	<0.001
	Day 14	2.78 ± 2.28	
Placebo	Day 1	5.44 ± 1.54	0.008
	Day 14	4.31 ± 2.14	

SD, standard deviation.

Seventeen dogs (94.4%) in the treatment group had a numerically lower PVAS score at Day 14 compared to Day 1. Twelve dogs (66.7%) meet the criteria for treatment success, and six dogs (33.3%) met the criteria for remission. Sixteen dogs (69.6%) in the placebo group had a reduction in PVAS scores at Day 14. Five placebo dogs (21.7%) met the criteria for treatment success, and five dogs (21.7%) met the criteria for remission. No difference in the remission rate was detected between the groups (P = 0.42).

**Figure 2:**
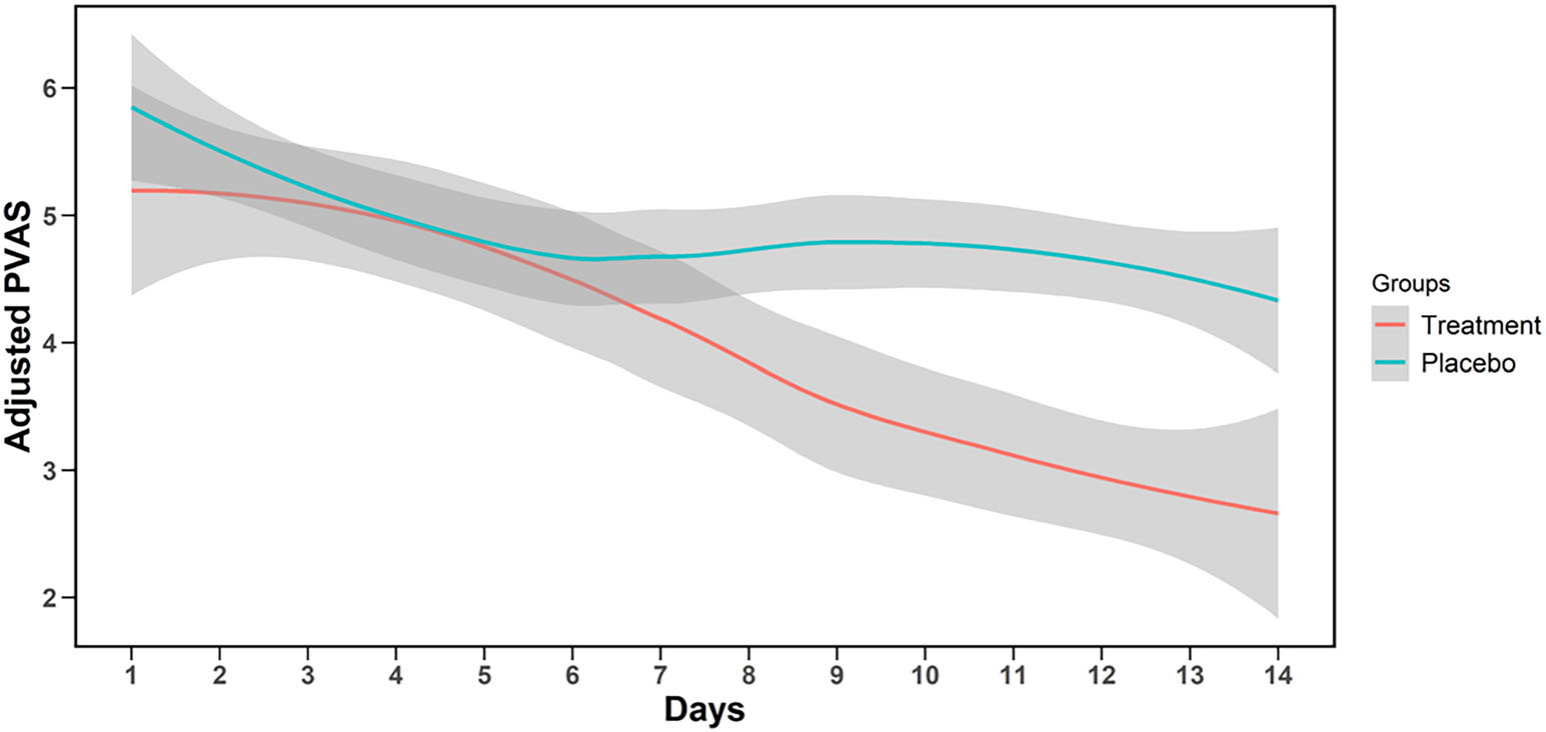
Treatment effect over time. Adjusted Pruritus Analog Visual Scale (PVAS) were calculated by subtracting the mean difference between the treatment and placebo groups at Day 1 from each subsequent day of treatment. Differences became significant at Day 9 (P = 0.01) and continued to be significant through Day 14.

A linear mixed effect model accounting for the variability in PVAS scores at Day 1 demonstrated that the treatment group showed a significant reduction compared to the placebo group starting at Day 9 (P < 0.001) and continuing for the duration of the trial (P ≤ 0.001 at each time point for Days 9–14) (Figure 2. Tables S1 and S2).

### Lesion scoring

**Figure 3:**
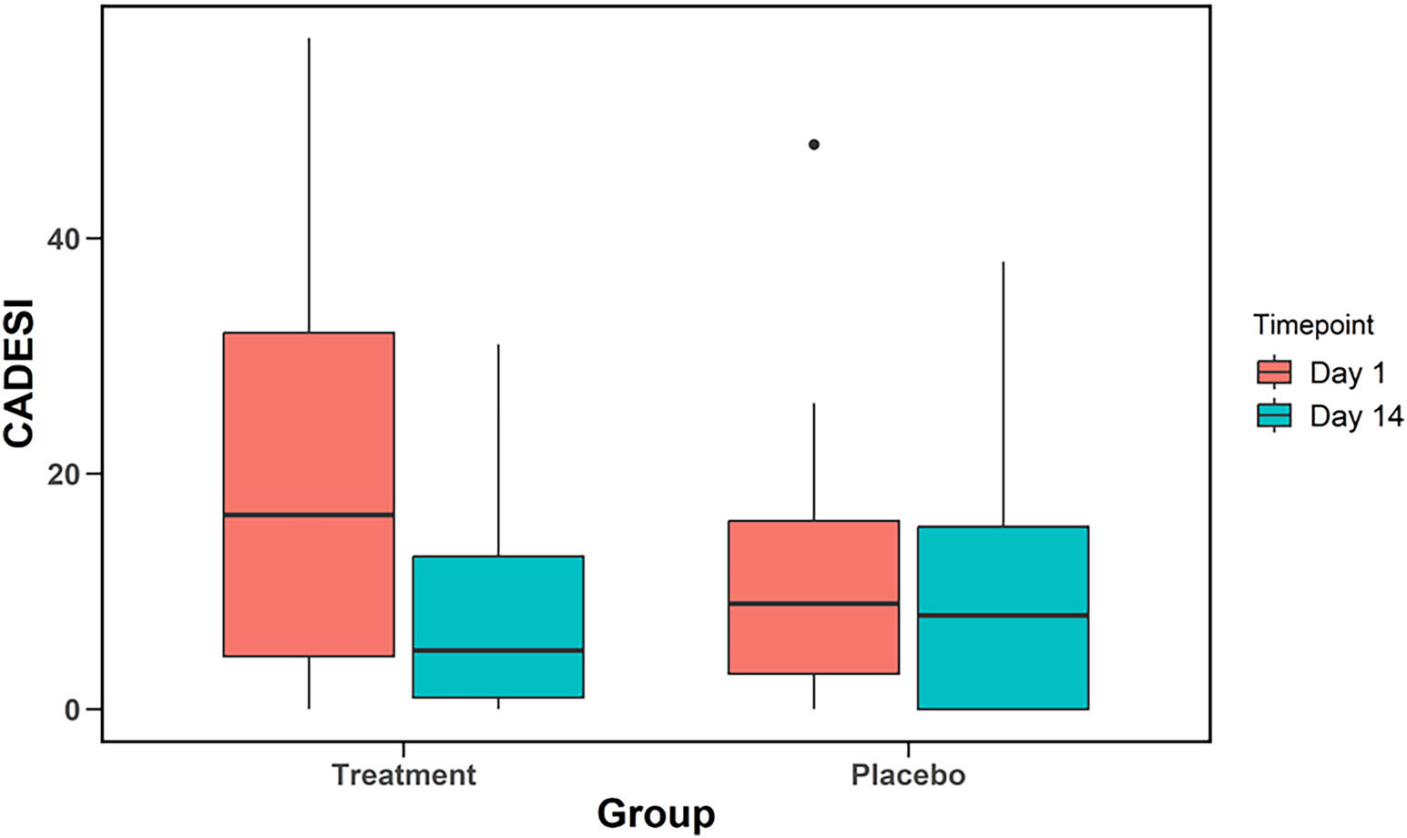
Comparison of pretrial (Day 1) and post‐trial (Day 14) lesion scores (Canine Atopic Dermatitis Extent and Severity Index – 4th Generation [CADESI‐4]) in the treatment and placebo groups. The treatment group saw a significant decrease (P = 0.001), whereas the change in the placebo group was not significant (P = 0.404).

At Day 1, the mean CADESI‐4 scores for the treatment and placebo groups were 19.33 ± 11.62 and 11.22 ± 11.28 respectively (P = 0.1). At Day 14, a significant improvement was identified in the treatment group 8.78 ± 9.39 (P = 0.001), but no effect was seen in the placebo group 9.91 ± 10.99 (P = 0.4) (Figure 3). On average, there was a 9.25‐point greater reduction in CADESI‐4 scores of the treatment group compared to the placebo group (P = 0.004).

**Figure 4:**
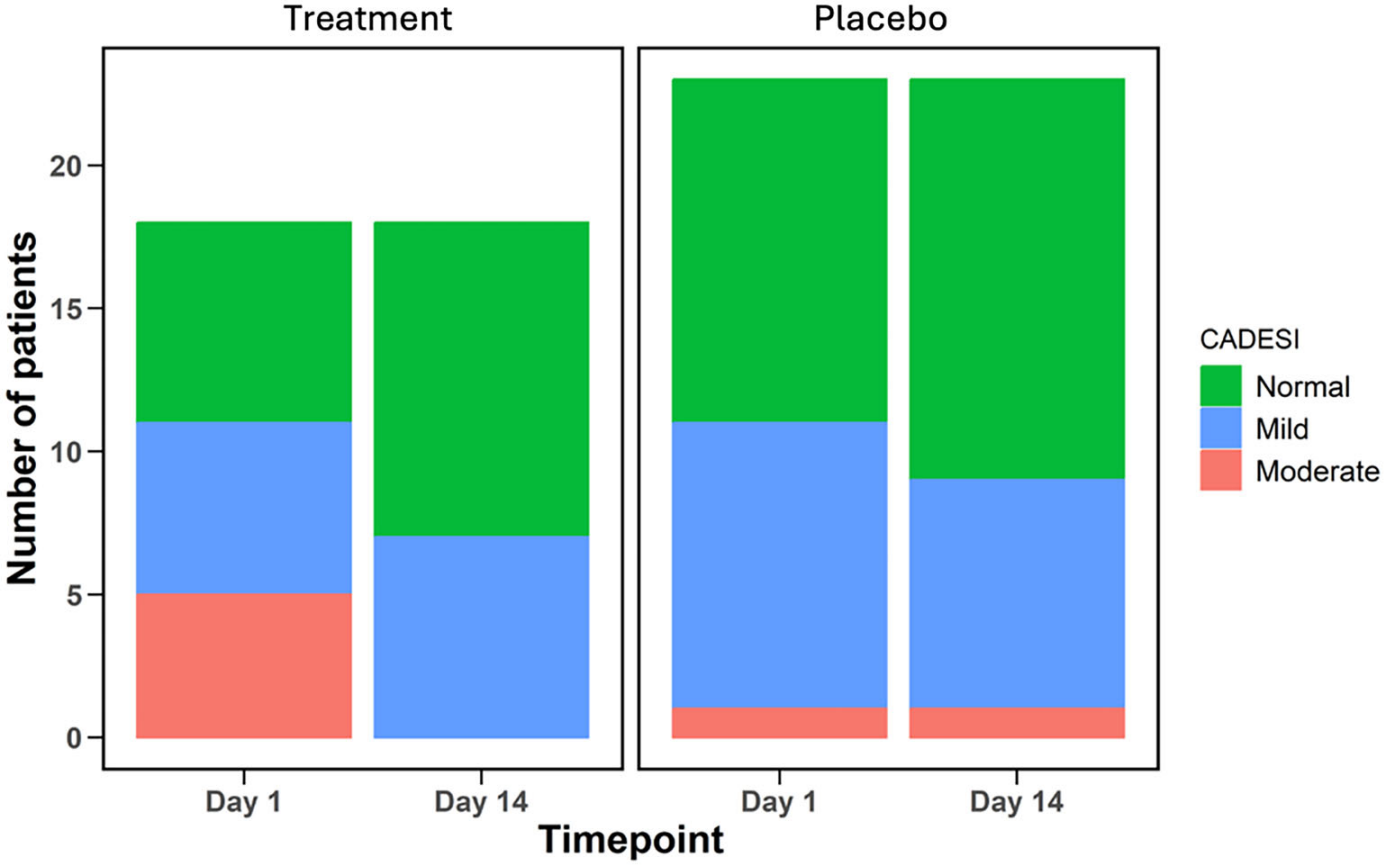
Distributions of Canine Atopic Dermatitis Extent and Severity Index – 4th Generation (CADESI‐4) categories within each group at both timepoints. All dogs in the treatment group saw improvement, with all moderately affected dogs reducing to mild or normal CADESI‐4 scores.

If CADESI‐4 scores are considered categorically (normal, mild, moderate, severe; Table 5; Figure 4), there is a very suggestive shift towards normal and mild cases in the treatment group (P = 0.05), but no such shift is seen in the placebo group (P = 0.08).

**Table 5:** Distribution of Canine Atopic Dermatitis Extent and Severity Index – 4th Generation (CADESI‐4) categories between patients in treatment and placebo groups

		CADESI‐4 category			
		Normal (<10)	Mild (10–34)	Moderate (35–59)	P value
Treatment	Day 1	7	6	5	
	Day 14	11	7	0	0.05
Placebo	Day 1	12	10	1	
	Day 14	14	8	1	0.83

The difference in distribution among categories after the study period is strongly suggestive of a difference in dogs in the treatment group, but not in the placebo group.

**Figure 5:**
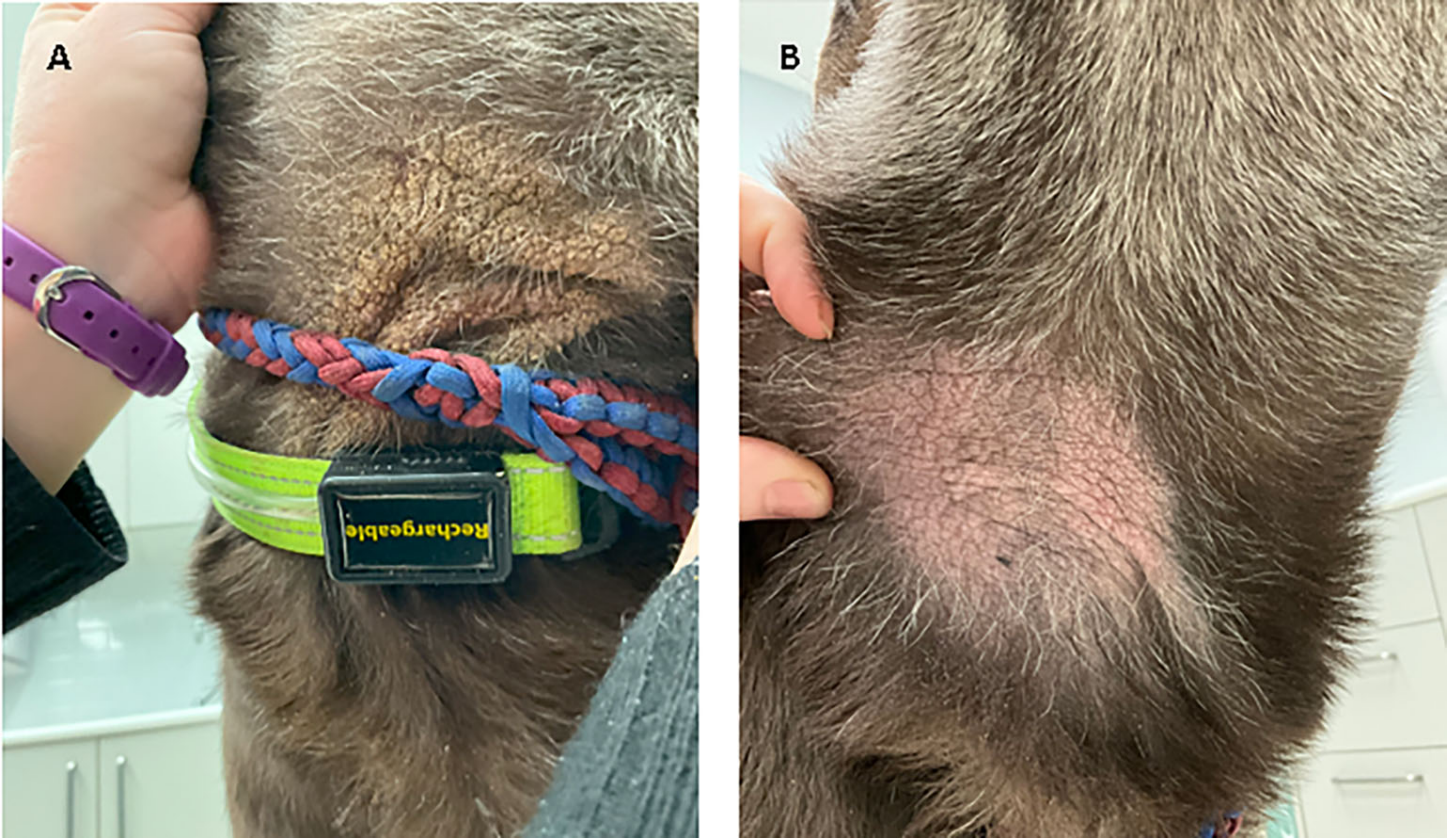
Improvement of a chronic lesion on the ventral neck of a dog, after 14 days in the treatment group. The owner‐reported that the lesions on this dog have been present for around 2 years and have been unresponsive to other treatments. At (A) Day 1, the lesion was lichenified, alopecic, rough on palpation and pruritic. Only occasional, extracellular cocci bacteria were found on cytology. At (B) Day 14, the skin was soft and supple, the crusts were no longer present and hair regrowth was starting to become apparent. Owners had reported an overall reduction in pruritus of 58%, resulting in a Pruritus Analog Visual Scale (PVAS) of 2.6/10 on Day 14. This is considered an improvement from severe to mild pruritus, approaching remission. Overall Canine Atopic Dermatitis Extent and Severity Index – 4th Generation (CADESI‐4) score was 57 (moderate) on Day 1 and had decreased to 25 (mild) by Day 14 (56.1% improvement).

Normal CADESI‐4 scores were calculated at both visits for 7/18 treatment dogs and 11/23 placebo dogs. When considering only dogs with abnormal CADESI‐4 scores, the remaining abnormal CADESI‐4 dogs in the treatment and placebo groups had a mean score of 29.8 ± 14.6 and 18.5 ± 11.1 respectively on Day 1 (P = 0.05). All dogs with abnormal CADESI‐4 scores in the treatment group (11/11, 100%) had lower numerical scores at the end of the study whereas only 5/11 (45.4%) abnormal placebo dogs had lower scores (Figure 5). Abnormal CADESI‐4 score dogs in the treatment group had a mean decrease in CADESI‐4 score of 16.3 ± 10.3 and dogs in the abnormal placebo group had an average decrease of only 1.4 ± 9.9 (P = 0.002). At the end of the study, 4/11 (36.4%) of mild/moderate treatment dogs and 3/12 (25%) of mild/moderate placebo dogs reduced to a normal CADESI‐4 score.

### Quality of life

At Day 1, there was no difference between the QoL scores of the treatment group (13.90 ± 8.17) compared to the placebo group (11.00 ± 4.30; P = 0.12). QoL scores improved in both groups by Day 14, with treatment group scores decreasing significantly to 7.5 ± 5.39 (P = 0.004) and the placebo group decreasing to 8.5 ± 4.22 (P = 0.09). The reduction in QoL scores in the treatment group was more than double the reduction in QoL scores in the placebo group (4.39 vs 2), and this finding was highly suggestive, but not significant (P = 0.06).

### Haematology and serum biochemistry

Complete blood counts and biochemistry were performed on Day 1 and 14. At each timepoint, dogs were coded as ‘normal’ (within reference range) or abnormal (above or below the reference range) for each parameter. All of the reported abnormal values were minimally out of range, and none were of clinical significance. There was no significant difference in the number of normal versus abnormal parameters between the start and end of the trial when comparing the treatment and placebo groups for either CBC (P = 0.83) or biochemistry (P = 1.00). Nearly all parameters for all dogs remained unchanged.

### Compliance, ease of use and tolerance

Owner compliance was extremely high, with 39/41 (95.1%) diary records for dogs completing the study indicating complete compliance with the trial protocol. Of the remaining two owners (both in the placebo group), one owner only missed a single day; the second owner missed two, nonconsecutive days in different weeks of the trial. Since these issues with noncompliance were nonconsecutive, and never more than once in a single week, these dogs were not excluded from any subsequent analyses.

No difference was detected between the treatment and placebo groups for ‘Ease of use’ (Day 7, P = 0.77; Day 14, P = 0.83) or ‘Dogs' attitude’ to cream application (Day 7, P = 0.42; Day 14, P = 0.7) during either week in the trial. At Day 14 75% of owners rated the treatment cream ‘very easy’ or ‘easy’. Owners' perception of their dogs' attitude showed that 71.4% of dogs were tolerant of, not bothered by or enjoyed the application of the cream.

### Adverse events

In the treatment group, one dog experienced vomiting on the first day, reported to have ingested cream from licking it off the skin where a visibly excessive amount of cream was applied. The owner was provided repeat instructions on proper application. A second dog experienced diarrhoea on Day 8. Both were reported to be transient, resolved by the following day without intervention, and both dogs completed the trial without any further adverse events. In the placebo group, one owner stopped using the cream on their dog's abdomen on Day 13 reporting the occurrence of bleeding and scabs.

## Discussion

In this double‐blind, placebo‐controlled, randomised clinical trial, the efficacy of a novel topical cream in relieving the clinical signs of pruritus and dermatological lesions in client‐owned dogs was evaluated. Treatment with the study cream was associated with a significant improvement in both owner‐scored pruritus and veterinarian‐scored lesions after 14 days of treatment. These results support the hypotheses that this topical product was efficacious at improving pruritus and reducing the severity of dermatological lesions. Additionally, this product was easy for owners to use, generally well tolerated by their dogs, improved the quality of life for both the dog and the owners and demonstrated minimal adverse events associated with treatment.

Dogs treated with the study cream showed a significant improvement in pruritus scores compared to the placebo. At the start of the study, the mean PVAS of dogs in both groups was consistent with previously published data suggesting a typical score of 5 to 6 out of 10 seen in dogs with chronic atopic dermatitis or adverse food reactions. Although the number of dogs that achieved clinical remission (PVAS ≤ 1.9) did not quite reach significance between the study groups, it should be noted that almost all treatment group dogs (94.4%) showed some level of improvement, and one third did achieve complete remission within the 2‐week period. This is comparable to clinical trials evaluating the efficacy of lokivetmab which showed 34.9%–37.9% of dogs achieved remission after 14 days.^10^, ^28^ In our study, 66.7% of dogs treated with the cream were considered to have achieved a treatment success in the reduction of pruritus, and this compares favourably to 77.0% of dogs that responded to lokivetmab in a retrospective study using the same metric.^25^ The mean owner PVAS after 14 days of treatment with lokivetmab or ciclosporin was reported as 3.3 and 4.6, respectively, compared to 2.78 seen here.^10^ The lack of an apparent plateau effect within the treatment period of this study (Figure 2) suggests that longer‐term use of this product may be associated with a continued response to treatment and the potential for additional dogs reaching clinical remission.

Given the study's focus on measuring the cream's effects on pruritus relief, the treatment duration was limited to two weeks. It was not expected that severe or chronic lesions would show complete resolution within a very short timeframe. Therefore, the severity of CADESI‐4 scores was not considered as part of the inclusion criteria. In the final study group, 44% of dogs had CADESI‐4 scores categorised as ‘normal’. This limited the ability to interpret the true potential of resolution for more severe lesions. Although the number of moderately affected dogs was small, all four dogs in the treatment group classified as having moderate lesion severity by the CADESI‐4 improved to the level of mild or normal during treatment. These results are similar to the improvement seen with lokivetmab in a similarly powered, small‐scale study reporting that 61.1% of dogs showed an improvement in CADESI‐4 ≥ 50% of baseline after four weeks, compared to 66.7% that reached this threshold in only two weeks in the current trial.^29^

Dogs treated with this cream achieved an average 44.0% reduction in CADESI‐4 scores, compared to 49.5%–51.5% of dogs treated with oclacitinib daily for two weeks, with or without the addition of hydrocortisone spray, respectively.^30^ This indicates that the cream may be as effective as other conventional therapies for dogs with chronic dermatitis‐related skin lesions. More animals with severely affected skin are needed to confirm these findings.

The mean improvement in dog‐QoL (52.4%) reported in the current study is similar to the improvements seen in dogs receiving unspecified ‘conventional’ treatments for CAD (dog‐QoL = 59.5%, owner‐QoL = 28.0%),^21^ or lokivetmab (dog‐QoL = 45%, owner‐QoL = 27%).^31^

Many dogs in the placebo group experienced improvement in their clinical signs of pruritus during treatment. The placebo cream is expected to have an emollient quality due to the waxes and oils used in the formulation. It was therefore expected that there would be some therapeutic benefit to the inactive ingredients in some dogs, simply from hydrating the flaking or irritated skin. It is also likely that at least some of the improvement seen in both groups would be due to the subjective nature of owner‐reported PVAS scores. The partial responsiveness to pruritus likely contributes to the improvement in QoL scores also seen in this group.

This topical cream was considered easy to use by owners and was generally well tolerated by most dogs. These results suggested that the topical cream was considered easy to administer by 75% of owners and was well tolerated by 71.4% of dogs. Another indicator of ‘ease of use’ was the very high compliance level of owners in this study. Of those that completed the trial, 93.1% of owners showed 100% compliance with daily treatments. Of the three dogs lost to follow‐up, only one owner declined to continue with the study due to the smell of the cream. Most other owners liked the smell of the cream. One possible limitation to the use of this product is the difficulty of application of a cream to areas of some dogs covered by very dense or long fur. Three participants that were otherwise eligible to enroll in this study declined to enroll, as they were concerned that they could not apply the cream to pruritic but heavily furred affected areas (data not shown).

The definitive diagnosis of pruritus not secondary to infectious pyoderma or ectoparasites in dogs is challenging to determine and covers a wide range of diagnoses with broad presenting signs with varying severity that changes over time.^5^, ^32^ Most dogs enrolled in this clinical trial were likely dogs with atopic dermatitis based on the histories provided and lesion distribution. A definitive diagnosis for atopic dermatitis was not considered necessary for inclusion, as the study objective was to evaluate changes in pruritus and skin lesions of noninfectious causes, independent of diagnosis. The subsequent inability to identify and definitively treat individual pathologies underscores the value of having a variety of treatments with different mechanisms of action and demonstrated performance in symptomatic improvement. A combination of costs, adverse effects, and owner compliance impact the efficacy of any one treatment. There is a growing trend amongst owners towards the use of complementary treatments and those perceived as ‘natural‐based’ products.^15^ The cream combines traditional active ingredients (zinc and salicylic acid) with well‐established dermatological benefits with the possible synergistic effects of botanical and herbal essential oils and extracts. Marigold (*Calendula officinalis*) has been reported to have wound healing, collagen‐enhancing and antierythematous effects, whereas Bergamot has been shown to have antioxidant, antinociceptive and anxiolytic effects; both of these ingredients have also been shown to have anti‐inflammatory and antimicrobial actions.^17^, ^33^ Zinc has been used as a therapeutic tool for centuries and is still often used in human and veterinary dermatological preparations. Zinc oxide has been shown to have antibacterial, anti‐inflammatory, anti‐irritant, antioxidant, moisturising and wound‐healing effects, as well as acting as a barrier to water loss and microbial adherence and penetration.^34^, ^35^, ^36^, ^37^

The toxic dose of zinc in dogs is poorly described^38^; however, toxicosis rarely results from the use of zinc oxide, as it is poorly ionisable after ingestion.^39^ One case study reported apparent zinc toxicoses in a 5.6 kg dog ingesting approximately 125 g of 15.25% w/w zinc oxide cream over 7 days.^40^ This would be the equivalent of ingesting over 750 g of the product trialled here, an extremely unlikely scenario given that this would require consuming multiple jars of the product. Excess ingestion of zinc oxide usually results in emesis rather than systemic effects.^39^ There are no published studies describing long‐term adverse effects associated with topically applied zinc oxide cream and statements about long‐term safety cannot be made based on our 14‐day trial.

Salicylic acid has been demonstrated to have anti‐inflammatory, antiseborrheic and antioxidant effects^41^, ^42^ and improves the hydrophilic capacity of keratin through a reduction in skin pH.^42^ It is commonly used in shampoo formulations for various dermatological conditions including canine pyoderma.^42^ The toxicity of salicylic acid in a leave‐on application, is not well documented in dogs. In people, toxicity occurs if 6% salicylic acid lotion is applied to >70% of the body two or more times per day.^43^ The product used in this study contains only 1.8% salicylic acid and is intended for use over small regions of the body, making toxicity highly unlikely. As with zinc oxide, there are no published studies describing long‐term use of topical salicylic acid, and statements made about long‐term safety cannot be made based on our 14‐day trial.

There is no evidence of significant acute adverse events associated with short‐term use of the cream tested in this trial. Of the few adverse events reported with treatment, all were transient, mild and resolved without veterinary intervention.

The therapeutic cream evaluated in this study was associated with improvement in pruritus and severity of skin lesions. These improvements were greater than those seen with the placebo cream. Additional studies in dogs with more severe skin lesions with the addition of histopathologic findings are needed to better characterise the benefit of this cream. It was easy for owners to use, well tolerated by dogs, improved quality of life and carried a low risk for adverse drug reactions.

## Conflicts of interest and sources of funding

The study was funded by Red Healer PTY Limited and through a grant received from the Australian Companion Animal Health Foundation. The authors declare no conflicts of interest for the work presented here.

## Supporting information


**Table S1.** Details of the changes in PVAS scoring between treatment and control groups for each day of the study adjusted for the difference in initial PVAS between groups. Differences become and remain significant from day 10 to the end of the study.

**Table S2.** Comparison of the difference between treatment and placebo each day using linear mixed‐effects model. Differences become significant on day 9 and continue to the end of the study period.

## Data Availability

The data that support the findings of this study are available on request from the corresponding author. The data are not publicly available due to privacy or ethical restrictions.
