# Supplementary material for: Topical cream with essential oils, zinc and salicylic acid reduces pruritus and skin lesion scores in pruritic dogs
Source: Aust Vet J. 2025 Dec 8;104(4):196–205. doi: 10.1111/avj.70048 (PMC13041744; doi:10.1111/avj.70048)
Supplement: Supplementary file 1 — Table S1. Details of the changes in PVAS scoring between treatment and control groups for each day of the study adjusted for the difference in initial PVAS between groups. Differences become and remain significant from day 10 to the end of the study. [file AVJ-104-196-s001.docx]

| Day | Difference in Means | CI | p-value |
| --- | --- | --- | --- |
| 2 | 0.08 | -0.84 – 0.99 | 0.870 |
| 3 | -0.55 | -1.47 – 0.36 | 0.234 |
| 4 | -0.44 | -1.36 – 0.47 | 0.343 |
| 5 | -0.36 | -1.27 – 0.56 | 0.442 |
| 6 | -0.62 | -1.53 – 0.29 | 0.183 |
| 7 | 0.03 | -0.88 – 0.94 | 0.949 |
| 8 | 0.30 | -0.61 – 1.22 | 0.517 |
| 9 | 0.91 | -0.00 – 1.83 | 0.051 |
| 10 | 1.06 | 0.15 – 1.98 | 0.023* |
| 11 | 0.98 | 0.06 – 1.89 | 0.036* |
| 12 | 1.04 | 0.13 – 1.96 | 0.025* |
| 13 | 1.17 | 0.26 – 2.08 | 0.012* |
| 14 | 1.22 | 0.31 – 2.14 | 0.009** |

**Table S1:** Details of the changes in PVAS scoring between treatment and control groups for each day of the study adjusted for the difference in initial PVAS between groups. Differences become and remain significant from day 10 to the end of the study.
