# Supplementary material for: Topical cream with essential oils, zinc and salicylic acid reduces pruritus and skin lesion scores in pruritic dogs
Source: Aust Vet J. 2025 Dec 8;104(4):196–205. doi: 10.1111/avj.70048 (PMC13041744; doi:10.1111/avj.70048)
Supplement: Supplementary file 2 — Table S2. Comparison of the difference between treatment and placebo each day using linear mixed‐effects model. Differences become significant on day 9 and continue to the end of the study period. [file AVJ-104-196-s002.docx]

| Day | Difference in Means | SE | P value |
| --- | --- | --- | --- |
| 1 | -0.44 | 0.53 | 0.400 |
| 2 | -0.52 | 0.53 | 0.324 |
| 3 | 0.11 | 0.53 | 0.835 |
| 4 | 0.00 | 0.53 | 0.995 |
| 5 | -0.09 | 0.53 | 0.869 |
| 6 | -0.18 | 0.53 | 0.739 |
| 7 | -0.47 | 0.53 | 0.369 |
| 8 | -0.75 | 0.53 | 0.159 |
| 9 | -1.36 | 0.53 | 0.011* |
| 10 | -1.51 | 0.53 | 0.005** |
| 11 | -1.42 | 0.53 | 0.008** |
| 12 | -1.49 | 0.53 | 0.006** |
| 13 | -1.61 | 0.53 | 0.003** |
| 14 | -1.67 | 0.53 | 0.002** |

**Table S2**: Comparison of the difference between treatment and placebo each day using linear mixed effects model. Differences become significant on day 9 and continue to the end of the study period.
